# A phylogenetically informed delineation of floristic regions within a biodiversity hotspot in Yunnan, China

**DOI:** 10.1038/srep09396

**Published:** 2015-03-30

**Authors:** Rong Li, Nathan J. B. Kraft, Jie Yang, Yuhua Wang

**Affiliations:** 1 Key Laboratory for Plant Diversity and Biogeography of East Asia, Kunming Institute of Botany, Chinese Academy of Sciences, Kunming 650201, China; 2 Department of Biology, University of Maryland, College Park 20742, USA; 3 Key Laboratory of Tropical Forest Ecology, Xishuangbanna Tropical Botanical Garden, Chinese Academy of Sciences, Mengla 666303, China; 4 Key Laboratory of Economic Plants and Biotechnology, Kunming Institute of Botany, Chinese Academy of Sciences, Kunming 650201, China

## Abstract

Traditional attempts to delineate floristic regions typically focus on species distributions, often ignoring the rich context that phylogenetic relationships can provide. In this study, we explore how phylogenetic relatedness, taxonomic composition, and regional phylogenetic structure change across a global biodiversity hotspot region, Yunnan, located in southwestern China. We propose a system of floristic regions within Yunnan by combining data on the distributions and phylogenetic relationships of 1,983 genera of native seed plants. We identified eight distinct floristic regions in Yunnan, which were grouped into two larger northern and southern geographical units. Phylogenetic relatedness was well correlated with taxonomic composition between floras in Yunnan. Across the Yunnan region we examined, the central Yunnan region shows the lowest level of spatial turnover in phylogenetic relationships and taxonomic composition of the floristic assemblages. Using null model analyses, we found evidence of nonrandom phylogenetic structure across the region, in which four areas show higher phylogenetic turnover than expected given the underlying taxonomic composition between sites. Our results show that the integration of phylogenetic information can provide valuable insight in floristic assessments, and help us to better understand the structure of a global biodiversity hotspot.

A central aim in floristic study is to classify groups of organisms into meaningful geographical units at different scales for the purpose of better understanding patterns of biodiversity^1^,^2^, analogous to the way in which taxonomy groups species into higher taxa. For example, based on the similarity of floristic assemblages and the degree of endemism, hierarchical floristic regions have been proposed for the world's flora^3^ and East Asian plants^4^. These floristic regions represent a fundamental categorization of the geographical organization of plant life on Earth, and thus provide spatially explicit frameworks for many broad-scale ecological and evolutionary studies^5^,^6^,^7^. Systems of floristic regions also have been used extensively for large-scale conservation analyses, for example to identify unique floristic assemblages^8^. Previous floristic region delimitations have become the foundation of much research in modern biogeography and are still in use today^9^,^10^, despite the fact that many are built on limited information (e.g., taxonomic composition, species distributions) and lack a statistical basis. A major drawback of these early works is that they fail to explicitly consider phylogenetic relationships among species, and therefore do not reflect the varied evolutionary history of floristic regions.

The use of phylogenetic information is increasingly common in ecological and biogeographical studies exploring the role of ecological and historical processes in shaping differences in species composition among communities and among floras^11^,^12^,^13^. A key step in the definition of floristic regions is quantifying the spatial turnover of species and phylogenetic composition between sites^14^,^15^. Analogous to taxonomic beta diversity, which measures change in species composition across space, phylogenetic beta diversity measures how phylogenetic relatedness among species change across space^16^. The past decade has witnessed rapid developments in the formulation of phylogenetic beta diversity metrics^17^,^18^,^19^.

Another important approach in the study of biogeographic regions is cluster analysis, which is a used to classify groups of similar objects^20^. This method has been successfully used to delineate biogeographic regions on continental^21^ and global scales^2^. The increasing availability of high-resolution data on species-level distributions and the ability to infer large phylogenetic trees now permits the delineation of phylogenetic-based biogeographical regions at scales that were previously impossible. For example, by integrating data on the distributions and phylogenetic relationships of the world's amphibians, birds, and mammals, a global map of zoogeographic regions was generated, which identify 20 distinct zoogeographic regions nested within 11 large realms^22^. However, few studies have attempted this for floristic regions at either a global or regional scale.

The Yunnan region forms a major part of the Indo-Burma biodiversity hotspot^23^, which lies to the east of the Himalaya mountain range. This region is located at a transitional zone with the Sino-Japanese floristic region in the east, the Sino-Himalayan floristic region in the west, and the Palaeotropic floristic region in the south^4^. Historically, Yunnan was divided into five floristic regions based on field surveys and expert opinion^24^, but a more recent analysis divides Yunnan into seven floristic regions based on the modelled distribution patterns of woody plant species^25^. However, this region has not been analyzed from a phylogenetic perspective, so we lack an understanding of the critical evolutionary dimension of floristic diversity in this area.

The goal of this study is to evaluate the relationship between taxonomic and phylogenetic beta diversity and to explore patterns of spatial turnover of Yunnan floristic assemblages from both a taxonomic and phylogenetic perspective, with the hope that the results can guide delimitation of phylogenetically-informed floristic regions in this area^2^,^15^,^22^. Specially, we address the following questions: 1) How closely related are patterns of phylogenetic and taxonomic dissimilarity in the Yunnan flora? 2) What are patterns of spatial turnover in species composition and phylogenetic composition between floras in Yunnan? 3) Does delimitation of floristic regions of Yunnan incorporating phylogenetic information agree with previous biogeographic treatments of the floristic region?

## Methods

### Study area

**Figure 1:**
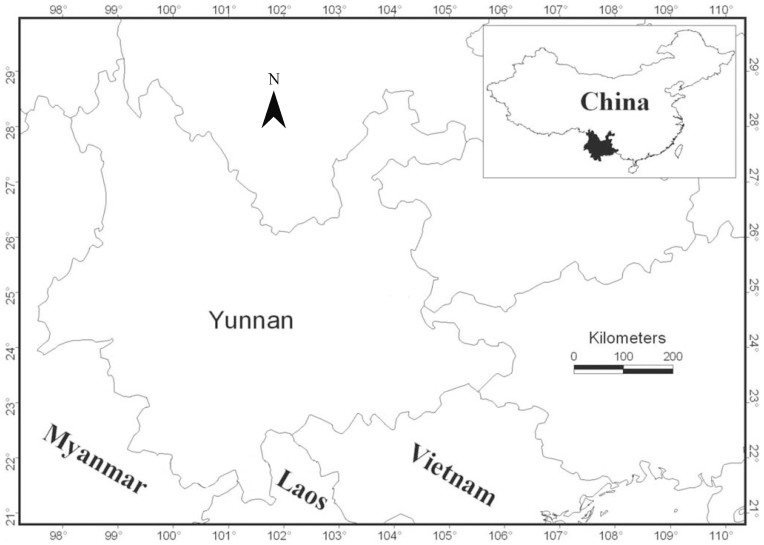
Location of Yunnan in China. The map was generated using DIVA-GIS 7.5.

**Figure 2:**
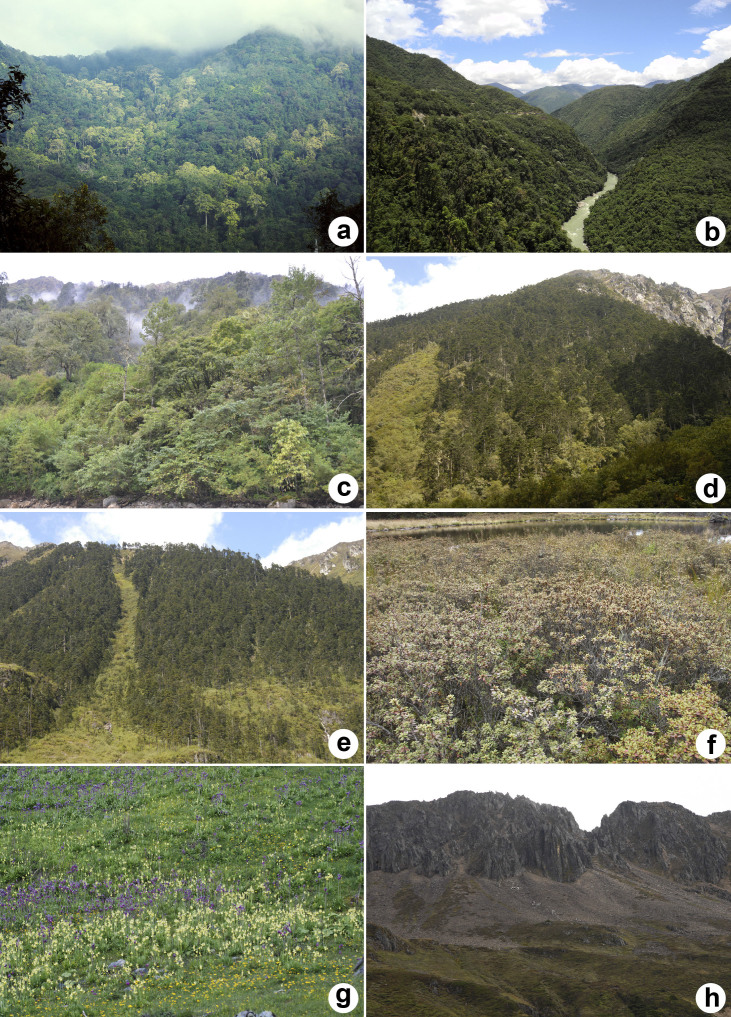
Images of vegetation in Yunnan. (a): tropical rain forest; (b): evergreen broad-leaved forest; (c): deciduous broad-leaved forest; (d): coniferous broad-leaved mixed forest; (e): coniferous forest; (f): alpine shrub; (g): alpine meadow; (h): alpine scree. The photos were taken by Rong Li.

Yunnan is located in southwestern China between 21°8′32″ – 29°15′8″ N and 97°31′39″ – 106°11′47″ E (Figure 1). It is a highland province with a terraced terrain stretching from the northwest (6,740 m a.s.l.) to the southeast (76 m a.s.l.)^24^. This region is one of the botanically most diverse terrestrial regions on Earth and contains nearly 18,000 plant taxa (3,008 genera and 433 families), which account for over 50% of China's overall floristic diversity^26^. The high levels of richness in the flora can be attributed to the geologic, topographic, and climatic diversity found within the area^27^. Acute topographical relief has created many peaks exceeding 4,000 m a.s.l. as well as deep valleys in this area. This topographic complexity presents substantial barriers to dispersal, and is thought to have been critical to the formation and development of the flora in this region^24^,^28^. The region also possesses a rich diversity of community types (Figure 2), including tropical rain forest, subtropical evergreen broad-leaved forest, temperate deciduous broad-leaved forest, temperate coniferous broad-leaved mixed forest, sub-alpine coniferous forest, alpine shrub, alpine meadow, and alpine scree^24^. Some parts of Yunnan have been identified as refugia during the Pleistocene^29^.

### Data sources

The present analyses utilize the Flora of Yunnan published in 21 volumes by Wu and his colleagues from 1977 to 2006^26^. The flora presents a comprehensive synonymized inventory of plant species in each county (N = 125) in Yunnan. In order to analyze the spatial changes in species composition and phylogenetic relatedness, the study area was divided into 125 county-level geographical units (the averaged for the county area is 3065.67 ± 1855.23 square kilometers; mean ± SD) (Supplementary Figures S1 and S2, Supplementary Table S1).

We determined the presence or absence of each seed plant genus in each county by extracting genus distribution information from Flora of Yunnan and generating a presence-absence genus matrix (see Supplementary Text S1 for details). Genera that are not native to Yunnan were excluded. We assigned each genus to a family, following the Angiosperm Phylogeny Group nomenclature^30^. A total of 77,582 records in 1,983 genera and 225 families were included in this study.

### Phylogeny construction

We constructed a phylogenetic tree by grafting the genera present in the study area onto a backbone phylogenetic hypothesis using the online program Phylomatic^31^. The backbone of the supertree was the Phylomatic tree version R20120829, based on Angiosperm Phylogeny Group's system^30^. Branch lengths in the supertree were adjusted to match clade age estimates reported by Wikstrom^32^ using the BLADJ algorithm implemented in the software Phylocom^33^. Given the scarcity of comprehensive time-calibrated phylogenies within families, we followed previous studies to treat genera as polytomies within families^34^.

### Beta diversity metrics

To quantify spatial turnover of Yunnan floristic assemblages in species composition and phylogenetic relationships between county-level floras, we calculated taxonomic beta diversity and phylogenetic beta diversity between localities. We used the Bray-Curtis index (*B*_bc_)^35^ and the mean nearest phylogenetic neighbor index (*D*_nn_)^33^ (see Supplementary Text S2 for equations) as our taxonomic and phylogenetic beta diversity measures, respectively, because they are both widely used and conceptually related^17^. The average taxonomic and phylogenetic dissimilarity between the focal region and all other regions were mapped to visualize the distinctiveness of each county-level assemblage in relation to the rest of the region.

Because phylogenetic beta diversity is likely to be related to taxonomic beta diversity, we used a null model analysis to test if county-level floras were more or less phylogenetically similar than expected by chance. A null distribution was generated for each county comparison by randomizing the names of the taxa on the phylogeny 999 times. This randomizes the phylogenetic beta diversity patterns between counties, while preserving the underlying taxonomic beta diversity differences between the samples. In each iteration, *D*_nn_ was calculated for each county comparison. The null distributions were used to calculate standardized effect sizes (SES_*D*_nn_), where the mean of the null distribution was subtracted from the observed dissimilarity *D*_nn_, and divided by the standard deviation of the null distribution^36^. Positive standard effect size measures indicate greater phylogenetic beta diversity than expected based on random sampling of the regional flora; negative standard effect sizes indicate the opposite. We then averaged the SES_*D*_nn_ values for each county to all other counties and mapped the results to illustrate patterns of regional turnover of phylogenetic composition.

Finally, we tested for correlations between species beta diversity, phylogenetic beta diversity and SES_*D*_nn_ using Mantel tests, which were used to analyze associations between distance matrices^37^. Permutation tests were applied to assess the significance of the correlation by randomizing the distance matrix 999 times. Analyses were performed in R 2.15.3 software using the 'vegan' and 'picante' packages.

### Cluster analysis

To delineate floristic regions of Yunnan by integrating data on the distributions and phylogenetic information, a hierarchical clustering was performed on the *D*_nn_ pairwise distances. Because the performance of clustering algorithms and linkage functions are largely determined by the characteristics of the input data^2^, we first tested the performance of five frequently used linkage functions in agglomerative hierarchical clustering. These functions were unweighted pair-group method using arithmetic averages (UPGMA), weighted pair-group method using arithmetic averages (WPGMA), Ward's method, single (SL) and complete linkage (CL), which were all implemented in the 'cluster' package in R. The validity of clustering results was evaluated using the cophenetic correlation coefficient^38^, which correlates pairwise distance from the leaves of a dendrogram to the encompassing node with the distances in the original distance matrix. This therefore represents a direct measure of how much of the original information is retained in the dendrogram^2^.

The choice of an adequate number of clusters is a longstanding issue in cluster analysis^39^,^40^. We determined the number of clusters and identified the floristic regions from the cluster results based on three criteria: (1) A reasonable number of cluster groups for floristic regions was determined following the method proposed by Halkidi^41^, which was implemented in the 'nbclust' package in R, (2) a floristic region is preferably represented by a group of contiguous sites, (3) each cluster that represents a floristic region needs to be a monophyletic clade in the dendrogram.

### Ordination

To provide a complementary non-hierarchical description of cluster relationships, floristic regions of Yunnan were also plotted in a two-dimensional ordination using a neighbor-joining algorithm by non-metric multidimensional scaling (NMDS) method^42^. NMDS is regarded as the most robust unconstrained ordination method for the depiction of overall turnover values within a matrix in a low-dimensional space^43^. We performed NMDS ordination using the *D*_nn_ pairwise distance for the cluster defined floristic regions in the 'vegan' library in the statistical software R. One hundred random starts were used to find a stable solution and to avoid local minima.

## Results

### Beta diversity

**Figure 3:**
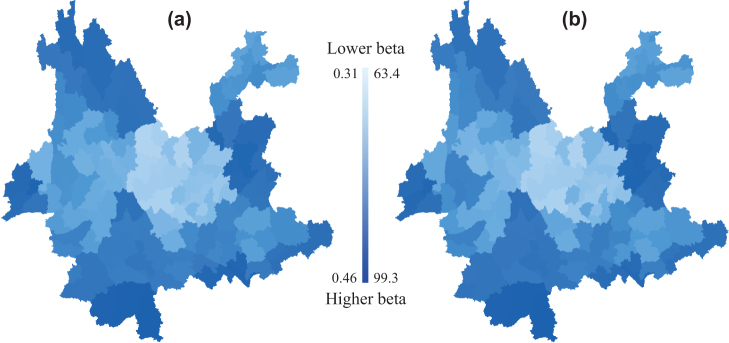
Maps of spatial turnover of Yunnan floristic assemblages in taxonomic composition (a) and phylogenetic composition (b). Color scale depicts the degree of taxonomic and phylogenetic turnover between the focal region and all other regions. The maps were generated using DIVA-GIS 7.5.

**Figure 4:**
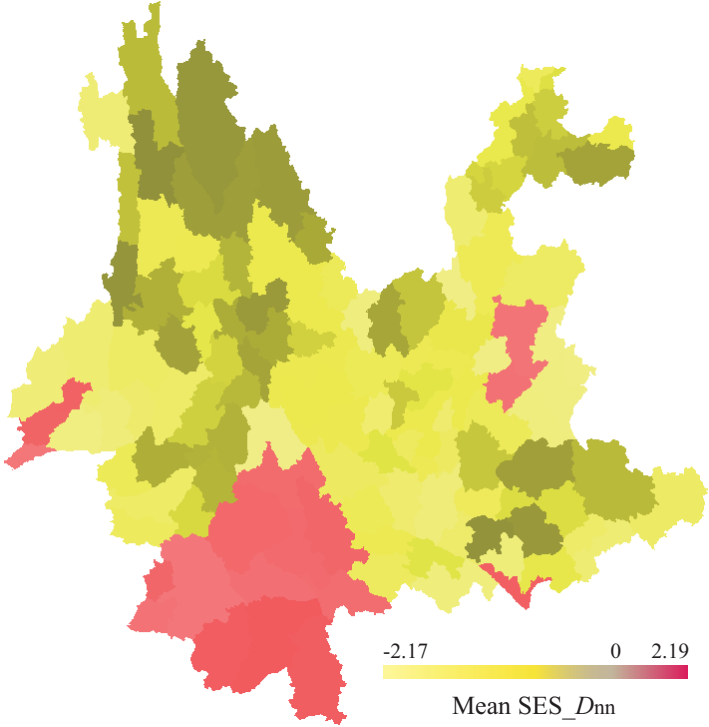
Patterns of regional turnover of phylogenetic relatedness between floras in Yunnan. The numbers are the average standardized effect sizes of phylogenetic beta diversity (SES_*D*_nn_) for each county to all other counties. Positive values indicate regions with higher phylogenetic turnover than expected, whereas negative values indicate the opposite. The map was generated using DIVA-GIS 7.5.

We found that in Yunnan phylogenetic beta diversity was strongly and positively correlated with taxonomic beta diversity (R^2^ = 0.99, *p* = 0.001). However, the standardized effect size of *D*_nn_ was not correlated with taxonomic beta diversity (R^2^ = 0.03, *p* = 0.192). The close correlation between phylogenetic dissimilarity and taxonomic dissimilarity in Yunnan's flora was also illustrated by the general spatial congruence in phylogenetic turnover and taxonomic turnover (Figure 3). The spatial turnover in taxonomic composition and phylogenetic composition of floristic assemblages is generally low in central Yunnan. The null model results of phylogenetic composition between floras in Yunnan detected four areas with higher phylogenetic turnover than expected given the underlying taxonomic composition between sites (Figure 4). In particular, southwestern Yunnan exhibits an evident regional turnover of higher phylogenetic composition.

### Floristic regions

The five clustering algorithms varied in performance for the *D*_nn_ pairwise distance (Table 1). Ward was the best performing clustering algorithm (cophenetic coefficient = 0.96; higher scores are better), followed by CL (cophenetic coefficient = 0.89), while single linkage performed worst (cophenetic coefficient = 0.78). Based on these results, we chose Ward's method.

**Figure 5:**
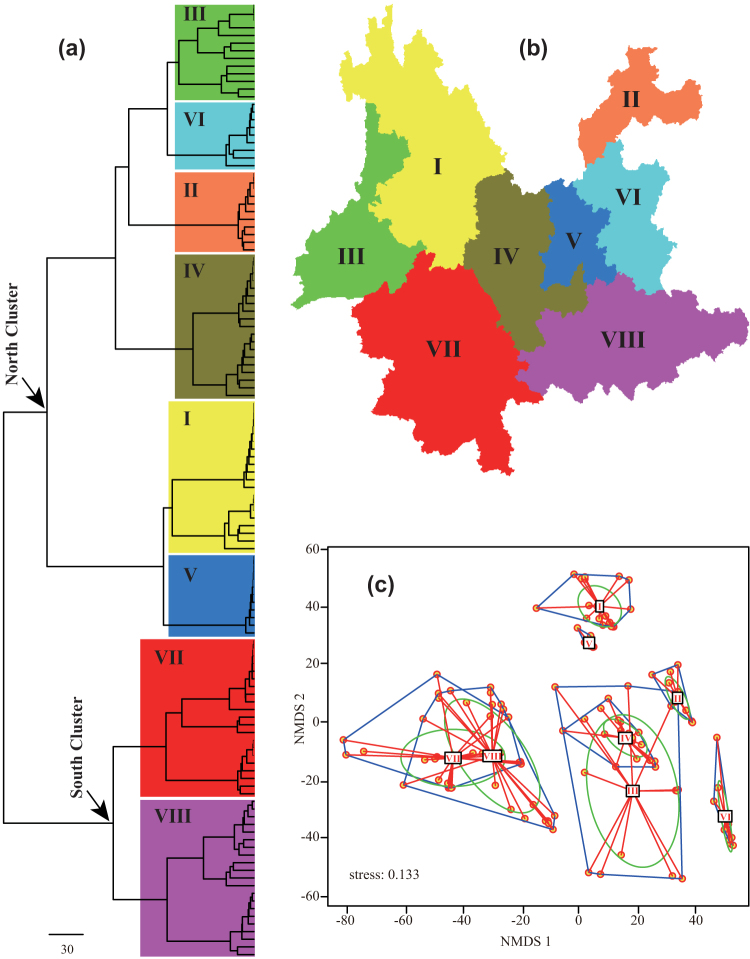
Dendrogram (a) and map (b) resulting from Ward hierarchical clustering and scatter plot (c) from non-metric multidimensional scaling (NMDS) two-dimensional ordination for floristic assemblages of Yunnan based on phylogenetic beta diversity distance matrices at the genus level. The eight distinct floristic regions are highlighted in the dendrogram with large colored rectangles and displayed in the map in different colors. The map was generated using DIVA-GIS 7.5.

Based on our three different criteria for setting a number of clusters, we selected eight as the optimal number of floristic regions for the floristic assemblages of Yunnan. These eight clusters fall into two larger clusters in the dendrogram (Figure 5, Supplementary Table S1), which follows a distinctive north to south pattern (Figure 5b), with southern regions VII and VIII form a single cluster (South Cluster) separate from the other six northern regions (North Cluster) (Figure 5a).

NMDS ordination led to satisfactory but not perfect projection of dissimilarity matrices into two-dimensional space indicated by relatively low stress values of 0.133 for the floristic regions of Yunnan (Figure 5c). Similar to the cluster analysis, a clear separation occurred between southern region assemblages (VII and VIII) and northern region assemblages (I to VI).

## Discussion

In this study, we have extended traditional floristic analyses based on taxonomic diversity to incorporate phylogenetic information. These analyses of phylogenetic dissimilarity of the entire floras use conceptual and analytical advances for quantifying phylogenetic beta diversity^44^,^45^. We have presented an analysis of spatial turnover of Yunnan floristic assemblages in species composition and phylogenetic composition to examine the correlations between taxonomic dissimilarity and phylogenetic dissimilarity in this area in order to test previous floristic regionalization from evolutionary perspective.

Our results indicate that taxonomic and phylogenetic turnover of the floristic assemblages in Yunnan are highly correlated. Moreover, related to the geographical location, central Yunnan shows the lowest level of spatial turnover in phylogenetic relationships and taxonomic composition of floristic assemblages (Figure 3). The flora in central Yunnan is a combination of species immigrated to this area from northwestern and southeastern Yunnan along the physiographic trending^46^. This process would lead to high floristic compositional similarity between central Yunnan and other regions. This may partly explain why floristic assemblages have on average a lower degree of spatial turnover in phylogenetic relatedness and taxonomic composition in central Yunnan than do other regions in Yunnan. Previous studies have shown this pattern. For example, Zhu showed that the floristic composition of central Yunnan has closely affinities with southern and northwestern Yunnan's floras, and is related to the flora of southern Yunnan mainly by tropical elements, especially tropical Asia and pantropic elements, while related to the flora of northwestern Yunnan mainly through temperate elements, especially north temperate and eastern Asia elements^47^.

Our study shows that the standardized effect size of *D*_nn_ was not correlated with taxonomic beta diversity. This implies that raw phylogenetic beta diversity is strongly influenced by the underlying patterns of species beta diversity. Moreover, we found evidence of nonrandom patterns of phylogenetic beta diversity across Yunnan's flora (Figure 4). For example, phylogenetic beta diversity in southwestern Yunnan is higher than expected, meaning that the taxa turnover between floras in this area occurs between distantly related taxa. Southwestern Yunnan has the largest tropical and subtropical forest cover in southwestern China, located at the northern edge of tropical Southeast Asia, the floristic assemblages in this area contain not only subtropical elements (e.g., Fagaceae, Lauraceae, Theaceae) but also some typical tropical elements (e.g., Dipterocarpaceae, Tetramelaceae, Lecythidaceae)^48^,^49^. In addition, several taxa in this area from clades that are endemic or narrowly distributed (e.g., Calophyllaceae, *Anogeissus*, *Gymnanthes*, *Polyosma*)^50^,^51^, which contributes to the high phylogenetic beta diversities measured.

The floristic regions that we identified were based on all the native seed plants genera which occur in Yunnan, which is the largest dataset available for this region today^26^. Our proposed eight floristic regions (Figure 5) are in broad agreement with previously published floristic division of Yunnan based on the qualitative evidence by expert^24^ and modelled distribution of woody plant species^25^. The main difference between the result of our analysis and previous works is that the western Yunnan region (region III) is a single floristic region, instead of being separated into regions I and VII in previous classifications^24^,^25^. The central Yunnan is a single unit in Wu's floristic region^24^ while here we divide it into three distinct regions (IV, V, and VI). The same result was also found in floristic regions of woody plants^25^. Species endemism is a key evidence to explain floristic region patterns^4^. We find that northwestern Yunnan region (region I) and southeastern Yunnan region (region VIII) are two of the 20 endemic centers in China^29^. The region I lies in the eastern fringe of the Tibetan Plateau, which includes rich neoendemic taxa (e.g., *Smithorchis*, *Formania*, *Hemilophia*) because of the uninterrupted uplift of the plateau since the late Neogene, which created a vast array of new habitats across wide elevational ranges (up to 5,000 m in this area) and stimulated allopatric and habitat differentiation, thus ultimately giving rise to the emergence of novel taxa^52^,^53^,^54^. The region VIII is located at the northern edge of tropical Southeast Asia, this region is characterized by high levels of palaeoendemism (e.g., *Malania*, *Bretschneidera*, *Lagarosolen*) because the relative tectonic and environmental stability in this area during the Tertiary may have facilitated the persistence of relict plant lineages^53^,^55^,^56^.

The major split of the main two clusters suggests the existence of a north-south split of the floristic region of Yunnan (Figure 5). The southern area might reflect adaptation to habitats of lowlands (below 1,400 m) affected by tropical climate whereas the northern area might be more influenced by subtropical climate on the habitats of middle elevations (1,300 – 2,200 m) and temperate to cold temperate climates in high mountains (above 1,900 m)^46^,^48^,^56^,^57^. In addition, the flora of Yunnan is largely influenced by the East Asian monsoon with higher summer rainfall and drought in winter except southern tropical region^58^ (see Supplementary Figure S3 for maps of temperature and precipitation). Geologically, the flora of northern Yunnan has evolved with the uplift of the Himalayas by gradual proliferation of mainly cosmopolitan and north temperate floristic elements, while the flora of southern Yunnan has evolved with extrusion of the Indochina block to Southeast Asia by the influence of mainly tropical Asian elements since the later Tertiary^27^,^47^,^59^. Such a historical and ecological difference is a potential explanation for the presence of the major north-south split of floristic regions of Yunnan. The results of this study show that southern cluster has a longer branch length than northern cluster (Figure 5a). Low rates of extinctions resulting from greater climatic stability in the southern tropical region could have contributed to this pattern by allowing species that belong to ancient clades to persist through time^51^,^56^,^60^.

The primary north-south divisions of floristic regions in Yunnan closely match with previous broad scale studies on vegetation communities. For instance, southern Yunnan is generally covered by tropical rain forest and tropical monsoon forest at lowlands, while northern Yunnan is covered by subtropical evergreen broadleaved forest and *Pinus yunnanensis* forest at middle elevations and covered mainly by temperate sclerophyllous forest and cold-temperate coniferous forest at high mountains^24^,^50^.

## Author Contributions

R.L. and N.J.B.K. conceived and wrote the paper. J.Y. analyzed the data. Y.W. provided the data. All authors reviewed the manuscript.

## Supplementary Material

Supplementary Information Supplementary Information

## Figures and Tables

**Table 1:** Cophenetic correlation coefficients for five different clustering methods performed on the mean nearest phylogenetic neighbor index (*D*_nn_) pairwise distances

Clustering algorithms	Cophenetic correlation coefficients
Unweighted pair-group method using arithmetic averages (UPGMA)	0.84
Weighted pair-group method using arithmetic averages (WPGMA)	0.85
Ward's method	0.96
Single (SL)	0.78
Complete linkage (CL)	0.89
