# Supplementary material for: A phylogenetically informed delineation of floristic regions within a biodiversity hotspot in Yunnan, China
Source: Sci Rep. 2015 Mar 30;5:9396. doi: 10.1038/srep09396 (PMC4377551; doi:10.1038/srep09396)
Supplement: Supplementary Information [file srep09396-s1.pdf]

A phylogenetically informed delineation of floristic regions within a biodiversity hotspot in Yunnan, China

Rong Li[1], Nathan J. B. Kraft[2], Jie Yang[3], Yuhua Wang[4]

[1] Key Laboratory for Plant Diversity and Biogeography of East Asia, Kunming Institute of Botany, Chinese Academy of Sciences, Kunming 650201, China

[2] Department of Biology, University of Maryland, College Park 20742, USA

[3] Key Laboratory of Tropical Forest Ecology, Xishuangbanna Tropical Botanical Garden, Chinese Academy of Sciences, Mengla 666303, China

[4] Key Laboratory of Economic Plants and Biotechnology, Kunming Institute of Botany, Chinese Academy of Sciences, Kunming 650201, China

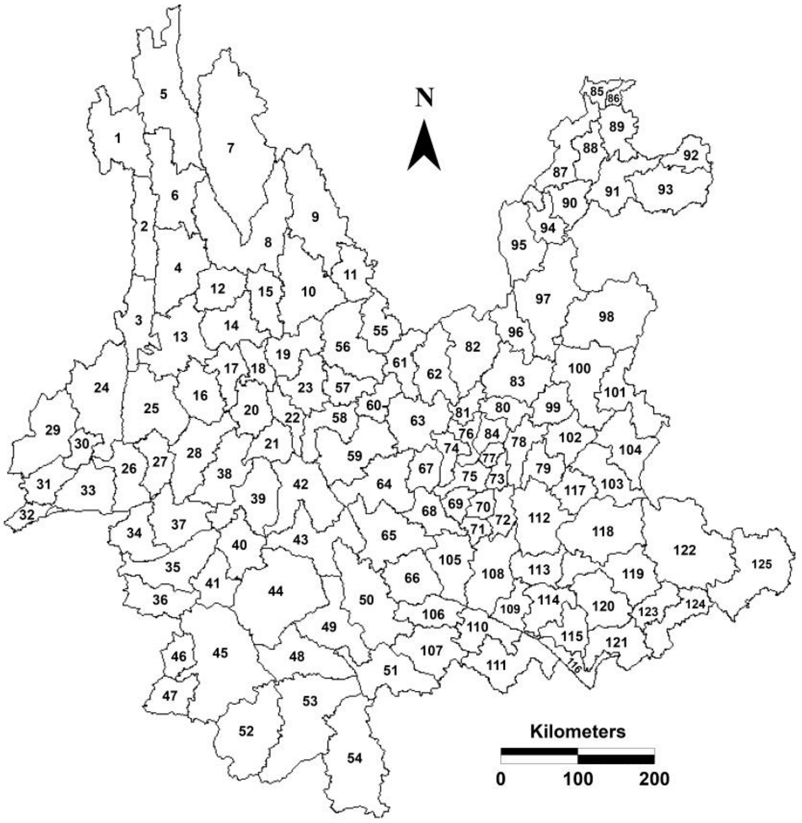

Figure S1 Map of administrative counties in Yunnan. The numbers correspond with the code in Supplementary Table S1, which indicate name of each county. The map was generated using DIVA-GIS 7.5.

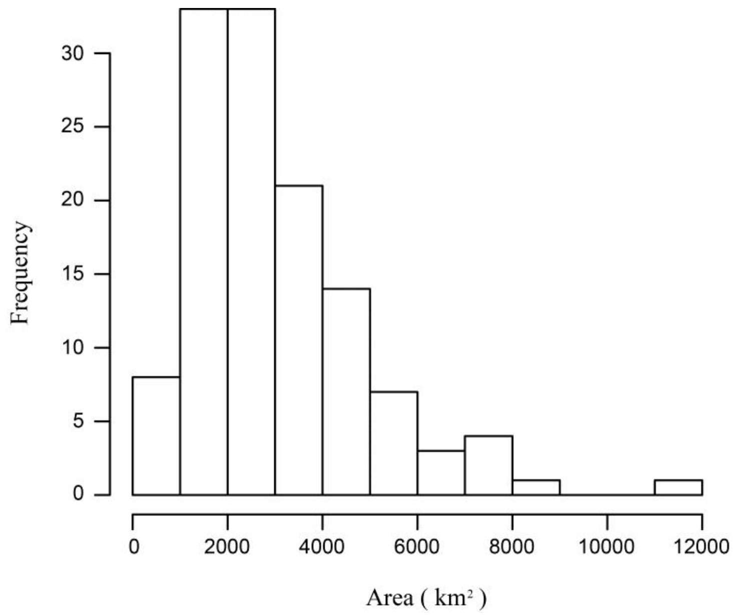

Figure S2 The area distribution for 125 counties in Yunnan.

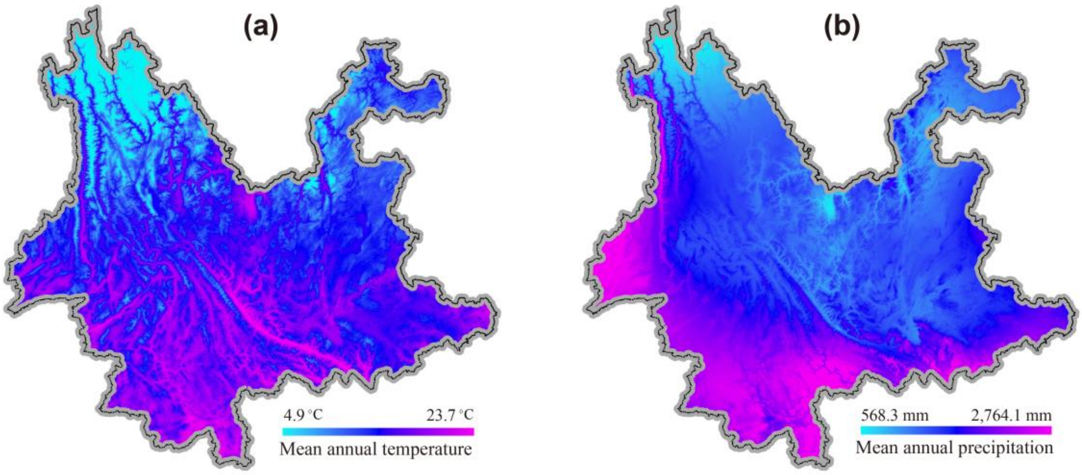

Figure S3 Maps of mean annual temperature (a) and mean annual precipitation (b) in Yunnan. Color scales depict the difference of temperature and precipitation among the regions in Yunnan. The maps were generated using ArcGIS 9.3.

Table S1 Area and floristic regions for each county in Yunnan. Code corresponds with the numbers in map of administrative counties (Supplementary Figure S1), which show the position of each county in Yunnan.

**Code**	**County**	**Area (**square kilometers**)**	**Floristic region**
1	Gongshan	4399.61	I
2	Fugong	2749.53	III
3	Lushui	3047.96	III
4	Lanping	4410.60	III
5	Deqin	7249.97	I
6	Weixi	4497.75	I
7	Zhongdian	11387.40	I
8	Lijiang	7504.44	I
9	Ninglang	5943.58	I
10	Yongsheng	4977.42	I
11	Huaping	2138.50	I
12	Jianchuan	2269.64	I
13	Yunlong	4422.51	I
14	Eryuan	2870.50	I
15	Heqing	2339.91	I
16	Yongping	2775.35	I
17	Yangbi	1877.42	I
18	Dali	1425.62	I
19	Binchuan	2549.33	I
20	Weishan	2177.60	I
21	Nanjian	1746.97	I
22	Midu	1501.44	I
23	Xiangyun	2434.62	I
24	Tengchong	5695.97	III
25	Baoshan	4834.70	III

26	Longling	2790.27	III
27	Shidian	1962.27	III
28	Changning	3787.73	III
29	Yingjiang	4333.33	III
30	Lianghe	1131.29	III
31	Longchuan	1841.17	III
32	Ruili	965.45	III
33	Mangshi	2891.04	III
34	Zhenkang	2541.13	VII
35	Gengma	3684.69	VII
36	Cangyuan	2484.45	VII
37	Yongde	3218.53	VII
38	fengqing	3350.15	VII
39	Yunxian	3624.80	VII
40	Lincang	2596.85	VII
41	Shuangjiang	2159.78	VII
42	Jingdong	4420.10	VII
43	Zhenyuan	4080.59	VII
44	Jinggu	7514.02	VII
45	lancang	8591.95	VII
46	Ximeng	1340.60	VII
47	Menglian	1913.79	VII
48	Simao	3862.14	VII
49	Puer	3670.14	VII
50	Mojiang	5253.40	VII
51	Jiangcheng	3460.14	VII
52	Menghai	5354.54	VII
53	Jinghong	6996.61	VII
54	Mengla	6821.65	VII

55	Yongren	2136.81	IV
56	Dayao	4080.05	IV
57	Yaoan	1717.37	IV
58	Nanhua	2290.90	IV
59	Chuxiong	4299.13	IV
60	Mouding	1481.60	IV
61	Yuanmou	2019.33	IV
62	Wuding	2984.23	IV
63	Lufeng	3517.10	IV
64	Shuangbai	3907.87	IV
65	Xinping	4231.58	IV
66	Yuanjiang	2814.46	IV
67	Yimen	1550.84	IV
68	Eshan	1923.07	IV
69	Yuxi	960.84	IV
70	Jiangchuan	821.89	IV
71	Tonghai	716.40	IV
72	Huaning	1255.84	IV
73	Chengjiang	719.13	IV
74	Anning	1275.84	V
75	Jinning	1339.74	V
76	Kunming	1107.84	V
77	Chenggong	507.10	V
78	Yiliang	1892.32	V
79	Lunan	1726.91	V
80	Songming	1387.85	V
81	Fuming	1030.30	V
82	Luquan	4205.74	V
83	Xundian	3561.27	V

84	Dongchuan	1055.38	V
85	Suijiang	780.19	II
86	Shuifu	425.93	II
87	Yongshan	2779.96	II
88	Daguan	1720.70	II
89	Yanjin	2008.37	II
90	Zhaotong	2170.43	II
91	Yiliang	2799.79	II
92	Weixin	1416.38	II
93	Zhenxiong	3701.95	II
94	Ludian	1509.44	II
95	Qiaojia	3195.11	II
96	Zhanyi	1901.54	VI
97	Huize	5855.56	VI
98	Xuanwei	6046.03	VI
99	Malong	1661.66	VI
100	Qujing	4257.34	VI
101	Fuyuan	3241.03	VI
102	Luliang	2014.25	VI
103	Shizong	2728.25	VI
104	Luoping	3064.62	VI
105	Shiping	2940.77	VIII
106	Honghe	2021.37	VIII
107	lvchun	3113.24	VIII
108	Jianshui	3820.31	VIII
109	Gejiu	1559.05	VIII
110	Yuanyang	2239.35	VIII
111	Jinping	3570.73	VIII
112	Mile	3901.52	VIII

113	Kaiyuan	1945.08	VIII
114	Mengzi	2143.39	VIII
115	Pingbian	1870.48	VIII
116	Hekou	1302.86	VIII
117	Luxi	1649.01	VIII
118	Qiubei	5038.34	VIII
119	Yanshan	3824.88	VIII
120	Wenshan	2982.07	VIII
121	Maguan	2663.10	VIII
122	Guangnan	7820.67	VIII
123	Xichou	1509.45	VIII
124	Malipo	2336.97	VIII
125	Funing	5287.89	VIII

Text S1 Data matrix.

The matrix format used to store and analyze data is the 'site-by-species' matrix. The site is a county-level geographical unit in our study. The sites are arranged in rows with the names of the rows being the names of the counties. The columns of matrix contain genera recorded in Flora of Yunnan with the column names as the unique names of genus. The values in the matrix are binary (1/0) indicating the presence or absence of each genus in each county. Totally, our data matrix has 125 rows and 1,983 columns.

Text S2 Beta diversity metrics.

The Bray-Curtis index (B_{bc}) is calculated as follows:

$$BC_{ij} = 1 - \frac{2C_{ij}}{S_i + S_j}$$

Where BC_{ij} is the Bray-Curtis dissimilarity between the sites i and j, C_{ij} is the sum of lesser values for those common species between both sites; S_i and S_j are the total number of species at each site.

The mean nearest phylogenetic neighbor index (D_{nn}) is calculated as follows:

$$D_{nn} = \frac{\sum_{i=1}^{n_{k_1}} \min\delta_{ik_2} + \sum_{j=1}^{n_{k_2}} \min\delta_{jk_1}}{n_{k_1} + n_{k_2}}$$

Where n_{k_1} represents the number of species in community k_1; n_{k_2} represents the number of species in community k_2; min δ_{ik_2} is the nearest phylogenetic neighbor distance between species i in community k_1 to all species in community k_2 and min δ_{jk_1} is the nearest phylogenetic neighbor distance between species j in community k_2 to all species in community k_1.
